# Multiparametric Analysis of Sniff Nasal Inspiratory Pressure Test in Middle Stage Amyotrophic Lateral Sclerosis

**DOI:** 10.3389/fneur.2018.00306

**Published:** 2018-05-02

**Authors:** Antonio Sarmento, Andrea Aliverti, Layana Marques, Francesca Pennati, Mario Emílio Dourado-Júnior, Guilherme Fregonezi, Vanessa Resqueti

**Affiliations:** ^1^PneumoCardioVascular Laboratory, Hospital Universitário Onofre Lopes, Empresa Brasileira de Serviços Hospitalares (EBSERH), Departamento de Fisioterapia, Universidade Federal do Rio Grande do Norte, Natal, Brazil; ^2^Dipartimento di Elettronica, Informazione e Bioingegneria, Politecnico di Milano, Milan, Italy; ^3^Ambulatório de Neurologia, Empresa Brasileira de Serviços Hospitalares (EBSERH), Departamento de Medicina Integrada, Universidade Federal do Rio Grande do Norte, Natal, Brazil

**Keywords:** amyotrophic lateral sclerosis, forced vital capacity, inspiratory muscle weakness, relaxation rates, respiratory subscore, sniff nasal inspiratory pressure

## Abstract

The relaxation rates and contractile properties of inspiratory muscles are altered with inspiratory muscle weakness and fatigue. This fact plays an important role in neuromuscular disorders patients and had never been extensively studied in amyotrophic lateral sclerosis (ALS). In this cross-sectional study, these parameters were investigated non-invasively through nasal inspiratory sniff pressure test (SNIP) in 39 middle stage spinal onset ALS subjects and compared with 39 healthy controls. ALS patients were also divided into three subgroups according to a decline in their percentage of predicted forced vital capacity (FVC_%pred_) as well as a decline in the ALS functional rating scale score and its respiratory subscore (R-subscore) in order to determine the best parameter linked to early respiratory muscle weakness. When compared with healthy subjects, middle stage ALS subjects exhibited a significantly lower (*p* < 0.0001) maximum relaxation rate and maximum rate of pressure development (MRPD), as well as a significantly higher (*p* < 0.0001) tau (τ), contraction time, and half-relaxation time. The results from receiver operating characteristic curves showed that MRPD (AUC 0.735, *p* < 0.001) and FVC_%pred_ (AUC 0.749, *p* = 0.009) were the best discriminator parameters between ALS patients with ≤30 and >30 points in the ALS functional rating scale. In addition, 1/2RT (AUC 0.720, *p* = 0.01), FVC_%pred_ (AUC 0.700, *p* = 0.03), τ (AUC 0.824, *p* < 0.0001), and MRPD (AUC 0.721, *p* = 0.01) were the parameters more sensitive in detecting a fall of three points in the R-subscore. On the other hand, MRPD (AUC 0.781, *p* < 0.001), τ (AUC 0.794, *p* = 0.0001), and percentage of predicted of SNIP (AUC 0.769, *p* = 0.002) were the parameters able to detect a fall in 30% of the FVC_%pred_ in middle stage ALS patients. The contractile properties and relaxation rates of the diaphragm are altered in middle stage spinal onset ALS when compared with healthy subjects. These parameters are able to discriminate between those middle stage ALS subjects with early decline in inspiratory muscle function and those who not.

## Introduction

Amyotrophic lateral sclerosis (ALS) is a rare neurodegenerative disorder characterized by progressive weakness of the skeletal and respiratory muscle (1). The median survival from first symptoms ranges from 2 to 4 years (2), and although respiratory insufficiency can be present in approximately 3% of patients (3, 4), it frequently emerges in the late phase of the disease representing the most frequent cause of death (1).

Global assessment scores, such as the ALS functional rating scale (5) (ALSFRS-R), is a useful and valid parameter in predicting survival in this population (6, 7) and has proved to be related to forced vital capacity (8) (FVC). Since respiratory function and muscle strength are of clinical importance and represent crucial factors influencing survival in ALS (9, 10), the monitoring of these parameters is essential during disease progression. The gold standard measurement of respiratory muscle strength involves the insertion of esophageal and/or gastric balloon catheters through the nose (11). However, the sniff nasal inspiratory pressure (SNIP) has been proposed as a non-invasive alternative method and proved to accurately reflect diaphragm strength (12) and global inspiratory muscle strength (13).

In ALS patients, the already weakened respiratory muscles are easily suitable to fatigue (14) and this fact may play an important role in the development of ventilatory failure (15). It has been demonstrated that the relaxation rate of inspiratory muscles is altered by the development of inspiratory muscle fatigue (16, 17) and that relaxation rates obtained from a maximal sniff accurately reflect those obtained from esophageal pressure (16, 18). Relaxation rates can be described in terms of maximum relaxation rate (MRR), half-relaxation time (1/2RT), and time constant of the pressure decay curve (τ, tau) after voluntary contraction of a muscle (16). Furthermore, the contractile properties of the diaphragm [namely, maximum rate of pressure development (MRPD) and contraction time (CT)] are also altered in fatigue and have been used as an index of the motor output of the respiratory center (19) as well as to assess inspiratory muscle function (11, 20, 21).

Apart from fatigue in healthy subjects (16–18, 22–24), physiological and/or disease-related changes in diaphragm relaxation have not been extensively investigated in ALS patients through the SNIP test. The present work aimed to non-invasively measure the relaxation rates and the contractile properties of the inspiratory muscles in ALS patients through SNIP test (1) in comparison to healthy subjects and (2) in relation to early respiratory symptoms in order to determine the best parameter linked to early respiratory muscle weakness. We hypothesized that these parameters are altered in ALS patients and can be indicators of inspiratory muscle weakness.

## Materials and Methods

### Subjects

This cross-sectional study was conducted according to the World Medical Association Declaration of Helsinki and approved by the Research Ethics Committee under number 1.344.512/2015. All individuals involved in the study signed an informed consent form.

We investigated 39 subjects with ALS (22 males), recruited from the Hospital Universirátio Onofre Lopes and diagnosed by a neurologist according to the *El Escorial* criteria (25) as “Probable or definite,” and 39 healthy controls (19 males). ALS subjects with cardiovascular, pulmonary, or other neurological diseases, as well as with bulbar dysfunction signs or tracheostomy were not included. Those who failed to perform the assessments or refuse to participate in the study were excluded.

Control group included self-reported age-matched healthy subjects with no history of cardiovascular, neurological or pulmonary diseases. Those with FVC and FEV_1_ <80% of predicted were excluded.

### Spirometry

Spirometry was performed using a Koko Digidoser spirometer (nSpire Health, Longmont, CO, USA) and carried out with the subjects positioned sitting on a chair with feet supported and trunk flexion of 90° according to the ATS/ERS guidelines (11). All values obtained were compared with absolute and percentage of predicted values for the Brazilian population (26).

### Respiratory Muscle Strength

Maximum inspiratory and expiratory pressures [maximum inspiratory pressure (MIP) and maximum expiratory pressure (MEP), respectively] and SNIP were measured using a digital manometer (NEPEB-Labcare, Belo Horizonte) with the subjects seated on a chair. MIP was measured starting from residual volume and MEP from total lung capacity, while SNIP was performed starting from functional residual capacity (FRC) (27). Data obtained were compared with previous reference values (28, 29), and the highest value of each test was considered for analysis.

### SNIP Curve Analysis

All subjects were asked to perform a short, sharp inspiratory effort through the nostrils with lips closed. Since some sniff parameters can be affected by changes in muscle length and the activity of expiratory muscles could interfere in the analysis, the sniff maneuvers were performed from FRC and a passive relaxation right after reaching the peak of pressure was requested (23, 30). At least 10 maximal sniffs, with an interval of about 30 s in between, were performed by all subjects. The following criteria were used to select those sniffs suitable for analysis: (1) sniff performed from FRC; (2) peak pressure maintained for less than 50 ms; (3) duration of the inspiratory effort less than 500 ms; and (4) sniff pressure waveform with smooth decay curve (16, 31).

**Figure 1:**
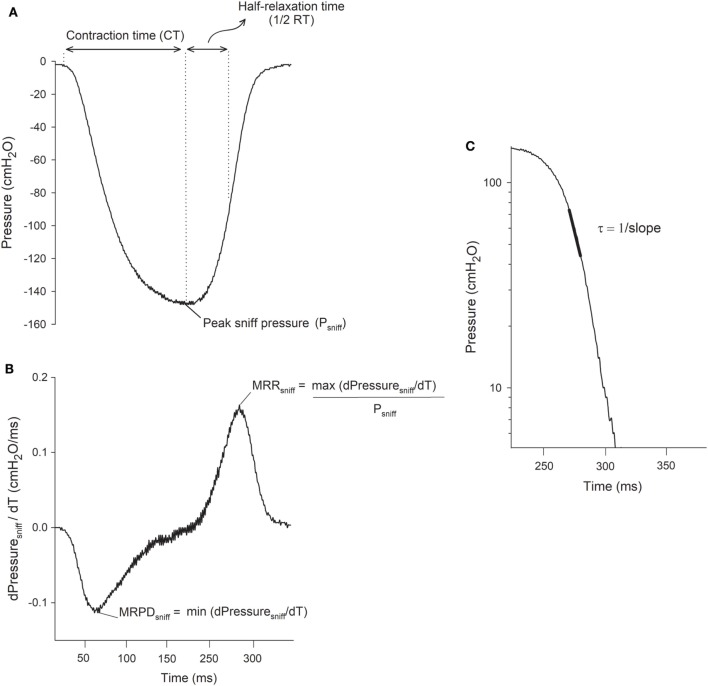
Representative tracings of the sniff nasal inspiratory pressure (SNIP) test and its parameters. **(A)** Tracings of SNIP change; peak sniff pressure (P_sniff_); time to reach P_sniff_, contraction time (CT); and half-time of the relaxation curve (1/2RT). **(B)** Derivative signal of sniff pressure (dPressure_sniff_/dT = cmH_2_O/ms); negative peak dP_sniff_/dT, maximum rate of pressure development (MRPD) positive peak dP_sniff_/dT normalized by P_sniff_, maximum relaxation rate (MRR). **(C)** Decay portion of the sniff pressure plotted on semilog scale vs. time (ms). Linear black portion indicates a single exponential function with a time constant, τ = 1/slope. cmH_2_O, centimeters of water.

Figure 1 shows the parameters derived from the SNIP test. From the sniff maneuver trace, CT and 1/2RT were calculated as the time to reach the peak pressure of the sniff and the half-time of the relaxation curve, respectively (32). MRPD, expressed as cmH_2_O·ms^−1^, was calculated as the negative peak of the first derivative of pressure–time curve (21, 33) while MRR, expressed as milliseconds^−1^, was defined as the positive peak of the first derivative of pressure–time curve normalized to the sniff peak pressure, in order to make contractions of different intensities comparable (18).

The time constant (τ), was also calculated. When the natural logarithm of pressure is plotted as a function of time, the lower 50–70% of the pressure decay follows a straight line (18, 34) (Figure 1C), indicating that the pressure follows a monoexponential decay with a time constant τ (τ = 1/slope). For the measurement of τ to be accepted, the correlation coefficient of the individual regression line (ln P vs. time) had to be ≥0.96 (35).

Sniff nasal inspiratory pressure curve analysis was performed by custom software developed in MATLAB (The MathWorksInc, Natick, MA, USA).

### Functionality and Stage of the Disease

Functionality was measured using the ALSFRS-R (maximum 48 points), validated for the Brazilian population (5), as well as its respiratory subscore (R-subscore) alone (36) (maximum 12 points). In addition, the stage of the disease was determined according to disease progression proposed by Roche et al. (37).

### Statistical Analysis

To statistical analysis, data from ALS subjects were divided into three subgroups, defined by the degree of decline of the (1) respiratory function (2, 38, 39) (≤70 and >70 of FVC_%pred_), (2) ALSFRS-R total score (≤30 and >30 points), and (3) R-subscore (≤9 and >9 points) (40, 41).

Data are expressed as median [25–75th percentile] unless otherwise stated. Normality and distribution of data were performed using Shapiro–Wilk test. Data between ALS and healthy subjects (intergroup analysis) were studied using the unpaired *t*-test or Mann–Whitney test for parametric and non-parametric data, respectively. One-way ANOVA or Kruskal–Wallis test was applied to compare subgroup with control group data and, in the event of statistical significance; Bonferroni’s or Dunn’s *post hoc* test was applied, respectively, to identify differences between groups.

To avoid type II error, the power of the study was calculated as well as effect sizes for all data. For parametric data, effect sizes were calculated using Cohen’s *d* for intergroup analysis and Cohen’s *f* for subgroup analysis (42). For non-parametric data, Cohen’s *d* was calculated for intergroup analysis according to Fritz et al. (43) and ε^2^ for subgroup analysis according to Tomczak and Tomczak (44) (see Supplementary Material).

Receiver operating characteristic (ROC) curves were calculated for SNIP parameters between middle stage ALS and healthy subjects, as well as between subgroups. The area under the curve (AUC) and its 95% confidence interval were calculated. Optimal cutoff point and its 95% confidence interval were also calculated for each parameter according to the Youden index (45).

Inferential data analysis was performed using GraphPad Prism^®^ software version 6.01. The power of the study and effect sizes were analyzed using G*Power software, version 3.1.9.2 (Kiel, Germany), and ROC curves were analyzed using MedCalc (Ostend, Belgium) version 14.8.1. For all statistical analysis, a *p*-value of <0.05 (two-sided) was considered as statistically significant.

## Results

Data related to diagnosis criteria, region of onset, clinical phenotype as well as the presence of familial ALS and cognitive impairment of all ALS included in the study are shown in Table S1 in Supplementary Material. Anthropometric, spirometric, respiratory muscle strength, and functionality data are shown in Table 1. ALS subjects were characterized by significant lower spirometric and respiratory muscle strength values. All ALS subjects were classified as middle stage. The mean ALSFRS-R score was 32.5 ± 8.8 (67.7 ± 18.3%), and the mean R-subscore was 10 ± 2 (83.3 ± 16.6) (see Table S2 in Supplementary Material).

**Table 1:** Characteristics of the subjects in relation to anthropometric data, absolute and predicted values of lung function, respiratory muscle strength, and functional capacity.

	Healthy	ALS	*p*
Subjects (*n*)	39	39	–
Age (years)	47.9 ± 11.1	52.9 ± 12.5	0.111
Height (ms)	1.63 ± 0.1	1.65 ± 0.1	0.500
Weight (kg)	69.6 ± 11	65 ± 13	0.100
BMI (kg/m^2^)	26.2 ± 5.6	23.9 ± 5.7	0.07
FVC (L)	3.79 ± 0.8	2.49 ± 1.06	<0.0001
FVC_%pred_	98.8 ± 10.7	63.1 ± 23.1	<0.0001
FEV_1_ (L)	3.10 ± 0.68	1.87 ± 0.83	<0.0001
FEV_1%pred_	98.8 ± 10.4	58.5 ± 21.9	<0.0001
FVC/FEV_1_	0.81 ± 0.04	0.76 ± 0.12	0.008
FVC/FEV_1%pred_	100.1 ± 4.4	94.3 ± 15.6	0.02
FEF_25–75%_	3.29 ± 0.87	1.86 ± 1.05	<0.0001
PEF (L/s)	6.41 ± 1.91	3.32 ± 2.19	<0.0001
SNIP (cmH_2_O)	103.3 ± 29.4	48.36 ± 27.04	<0.0001
SNIP_%pred_	100.4 ± 24	47.2 ± 24.7	<0.0001
MIP (cmH_2_O)	105 ± 27.45	48.1 ± 22.50	<0.0001
MIP_%pred_	103.3 ± 20.9	48.4 ± 22.7	<0.0001
MEP (cmH_2_O)	125.4 ± 36.46	58.46 ± 31.92	<0.0001
MEP_%pred_	121.8 ± 31.9	56.6 ± 32.1	<0.0001
ALSFRS-R	–	32.5 ± 8.8	–
Respiratory subscore	–	10 ± 2	–

*Data presented as mean ± SD*.

*FVC, forced vital capacity; FEV_1_, forced expiratory volume in the first second; FEV_1_/FVC, ratio of forced expiratory volume in the first second to forced vital capacity; FEF_25–75%_, forced expiratory flow at 25–75%; PEF, peak expiratory flow; MIP, maximum inspiratory pressure; MEP, maximum expiratory pressure; SNIP, sniff nasal inspiratory pressure; ALSFRS-R, amyotrophic lateral sclerosis functional rating scale-revised; %pred, percentage of predicted; cmH_2_O, centimeters of water*.

**Figure 2:**
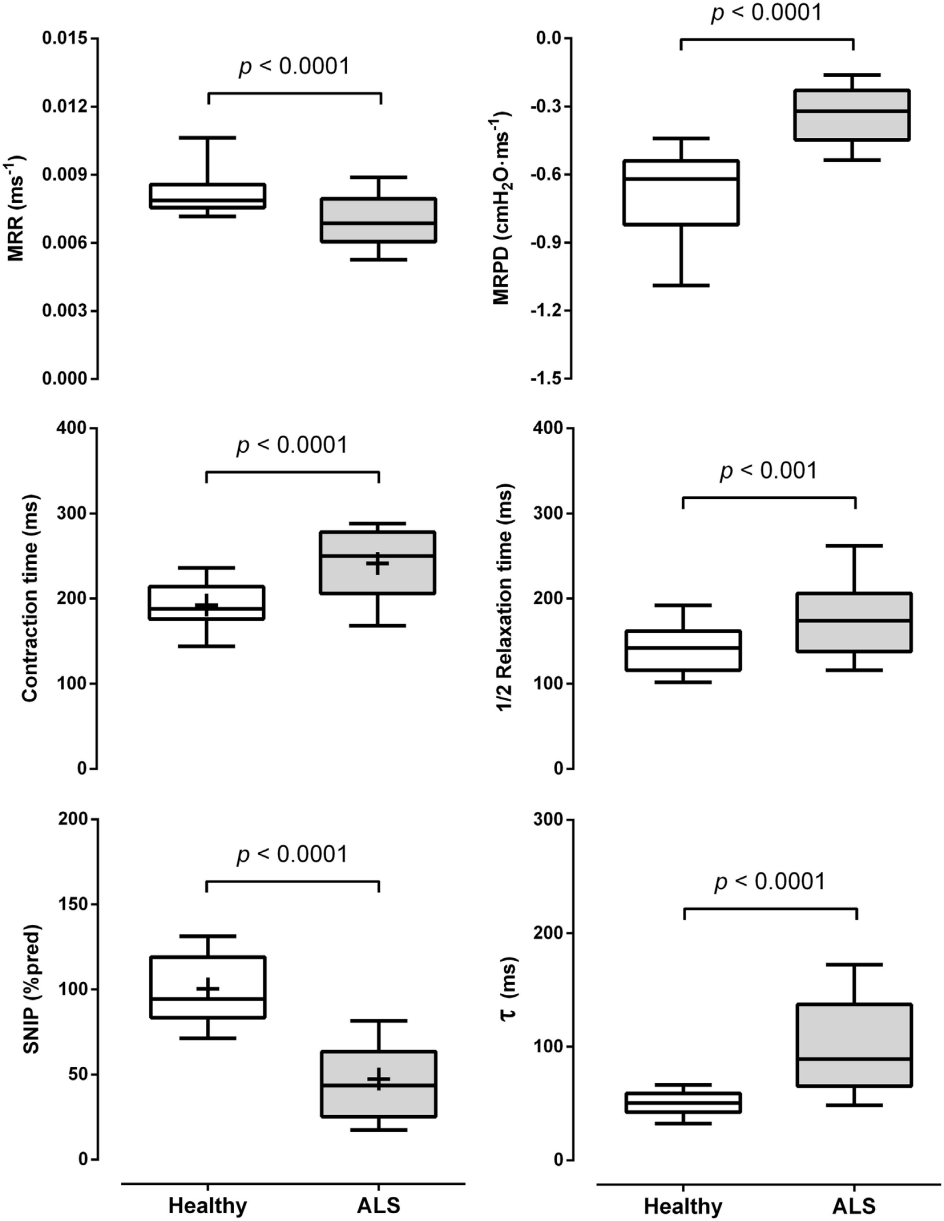
Data are shown as median [25–75th percentile]. Comparisons between the parameters obtained from the sniff nasal inspiratory pressure (SNIP) curve [maximum relaxation rate (MRR), maximum rate of pressure development (MRPD), contraction time, half-relaxation time (1/2RT), and tau (τ)] and percentage of predicted of the SNIP test (SNIP_%pred_) between amyotrophic lateral sclerosis (ALS) and healthy subjects. cmH_2_O: centimeters of water; +, mean for parametric analysis.

All parameters extracted from the sniff curve were significantly different between ALS and healthy subjects. A significantly lower MRR (*p* < 0.0001, Cohen’s *d* = 0.44) and MRPD (*p* < 0.0001, Cohen’s *d* = 0.71) were found in ALS subjects, as well as a higher CT (*p* < 0.0001, Cohen’s *d* = 1.21), 1/2RT (*p* < 0.001, Cohen’s *d* = 0.42), and τ (*p* < 0.0001, Cohen’s *d* = 0.64) (Figure 2).

A *post hoc* analysis considering a *p*-value of <0.01 and the calculated effect size for τ between ALS and healthy subjects (Cohen’s *d* = 0.64) showed a statistical power (1 − β) = 0.99 for this study.

### ALSFRS-R, R-Subscore, and FVC_%pred_ Subgroups

As shown in Table 2, all subgroups of ALS subjects presented a lower FVC_%pred_, SNIP_%pred_, MRR, and MRPD and higher CT, 1/2RT, and τ when compared with healthy subjects. However, subjects with functional capacity ≤30 (13 subjects) exhibited significantly lower values of FVC_%pred_ when compared to ALS subjects with >30 points; and those with ≤9 (14 subjects) presented a significantly lower FVC_%pred_ as well as higher 1/2RT and τ values when compared to those with >9 points. On the other hand, when ALS subjects were classified according to FVC_%pred_, those with <70% exhibited significantly higher τ and lower SNIP_%pred_ values when compared to ALS with values >70%.

**Table 2:** Relaxation rates and contraction properties of the diaphragm extracted from the SNIP curve of healthy and ALS subgroup subjects.

	Healthy	ALS								
		ALSFRS-R			Respiratory subscore			FVC_(%pred)_		
		>30 points	≤30 points	ES	>9 points	≤9 points	ES	>70%	≤70%	ES
Subjects (*n*)	39	26	13	–	25	14	–	15	24	–
MRR (ms^−1^)	0.0078 [0.0075 to 0.0085]	0.0069 [0.0061 to 0.0074]*	0.0065 [0.0043 to 0.0083]*	0.19^a^	0.0071 [0.0063 to 0.0079]*	0.0063 [0.0047 to 0.0079]*	0.21^a^	0.0071 [0.006 to 0.008]*	0.0067 [0.006 to 0.008]*	0.19^a^
MRPD (cmH_2_O·ms^−1^)	−0.620 [−0.821 to −0.540]	−0.348 [−0.470 to −0.272]*	−0.250 [−0.318 to −0.196]*	0.55^a^	−0.357 [−0.480 to −0.265]*	−0.250 [−0.344 to −0.187]*	0.55^a^	−0.460 [−0.535 to −0.280]*	−0.255 [−0.357 to −0.196]*	0.59^a^
τ (ms)	50.4 [42.3 to 58.8]	78.6 [57 to 121.1]*	127.7 [76.5 to 157.5]*	0.44^a^	69.6 [56 to 102.5]*	128.4 [101 to 177]*^,^^††^	0.50^a^	65.4 [55.8 to 85.9]*	111 [83.7 to 158]*^,^^†††^	0.84^b^
1/2 RT (ms)^c^	142 [116 to 162]	174 [135 to 206]*	174 [151 to 204]*	0.42^b^	160 [131 to 194]*	191 [169 to 267]*^,^^††^	0.57^b^	168 [132 to 190]*	181 [158 to 207]*	0.46^b^
CT (ms)	188 [176 to 214]	229 [197 to 274]*	268 [230 to 281]*	0.28^a^	220 [197 to 277]*	266 [244 to 278]*	0.30^a^	254 [210 to 282]*	241 [201 to 276]*	0.56^b^
SNIP (%pred)^c^	94.4 [83.5 to 119]	47.4 [24.8 to 75.2]*	39.2 [26.2 to 51.1]*	1.98^b^	46.7 [24.4 to 76.7]*	40.9 [25.9 to 56.8]*	1.13^b^	61 [38.3 to 81.5]*	38 [23.5 to 46.6]*^,^^†††^	1.17^b^
FVC (%pred)^c^	97.5 [91.5 to 107.5]	68.3 [54.3 to 82.4]*	41.7 [32 to 64.9]*^†^	0.97^b^	70.6 [51.3 to 82.8]*	54.7 [32.2 to 65.3]*^,^^††^	1.01^b^	–	–	–

*Values are shown as median [25th–75th percentile]*.

*ALSFRS-R, amyotrophic lateral sclerosis functional rating scale-revised; MRR, maximum relaxation rate: MRPD, maximum rate of pressure development; τ, tau; 1/2RT, half-relaxation time; CT, contraction time; SNIP, sniff nasal inspiratory pressure; FVC, forced vital capacity; ES, effect size; %pred, percentage of predicted; cmH_2_O, centimeter of water; ALS, amyotrophic lateral sclerosis*.

*<0.001 compared with healthy.

*^†^<0.001 compared with >30*.

*^††^<0.001 compared with >9*.

*^†††^<0.001 compared with >70%*.

*^a^Epsilon squared*.

*^b^Cohen’s f*.

*^c^Parametric data distribution*.

### ROC Analysis

**Figure 3:**
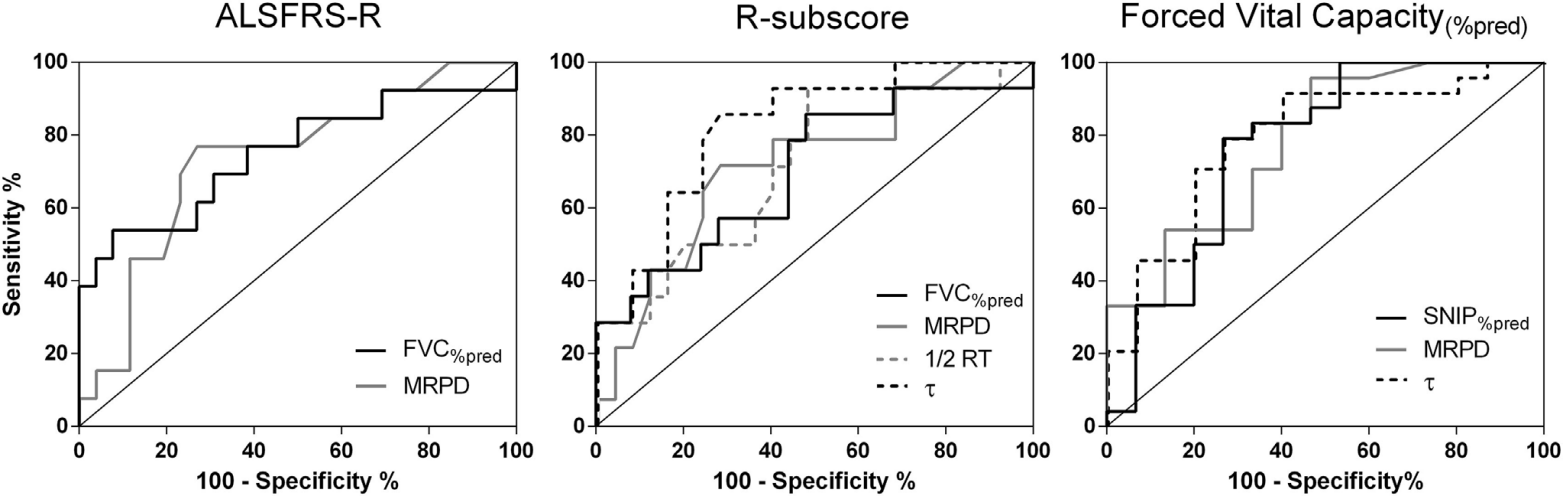
Receiver operating characteristic curves of sniff nasal inspiratory pressure (SNIP) parameters that showed to be statistically significant in middle stage ALS subjects according to a decline in the percentage of predicted forced vital capacity (FVC) and in the ALSFRS-R score and R-subscore.

Since SNIP_%pred_ is one of the respiratory prognostic markers mostly considered in ALS (38, 46), this parameter was also included in the ROC analysis. As shown in Table 3, all sniff parameters were significantly able to discriminate between ALS and healthy. Of these, MRPD was the parameter with the highest AUC. When dividing the ALS subjects between those with ALSFRS-R score ≤30 and >30, only the MRPD and FVC_%pred_ were statistically significant (Table 4). However, when taking into account the subdivision between those ALS with R-subscore ≤9 and >9 points, MRPD, 1/2RT, τ, and FVC_%pred_ showed to be statistically significant (Table 5). On the other hand, MRPD, τ, and SNIP_%pred_ parameters were statistically significant when subjects were classified according to FVC_%pred_ classification (Table 6; Figure 3).

**Table 3:** Receiver operating characteristic analysis between healthy and ALS subjects.

Healthy and ALS					
	AUC (95% CI)	Optimal cutoff (95% CI)	Sensitivity (%)	Specificity (%)	*p*
MRR (ms^–1^)	0.755 (0.645 to 0.845)	0.0073 (0.0068 to 0.0073)	66.67	89.74	<0.0001
MRPD (cmH_2_O ms^–1^)	0.916 (0.830 to 0.967)	−0.420 (−0.540 to −0.398.5)	74.36	97.44	<0.0001
τ (ms)	0.874 (0.779 to 0.938)	66 (53.7 to 79.8)	74.36	89.74	<0.0001
1/2 RT (ms)	0.743 (0.631 to 0.835)	154 (120.9 to 164)	71.79	71.79	<0.0001
CT (ms)	0.795 (0.688 to 0.878)	215 (202 to 262)	69.23	82.05	<0.0001
SNIP (%pred)	0.936 (0.856 to 0.979)	81.5 (73.7 to 81.5)	92.31	84.62	<0.0001
FVC (%pred)	0.911 (0.825 to 0.964)	81.1 (67.9 to 83.7)	79.49	97.44	<0.0001

*AUC, area under curve; CI, confidence interval; MRR, maximum relaxation rate; MRPD, maximum rate of pressure development; τ, tau; 1/2RT, half-relaxation time; CT, contraction time; SNIP, sniff nasal inspiratory pressure; FVC, forced vital capacity; ES, effect size; %pred, percentage of predicted; cmH_2_O, centimeter of water; ALS, amyotrophic lateral sclerosis*.

**Table 4:** Receiver operating characteristic analysis between ALS subjects classified according to a decrease in the ALSFRS-R scale score.

ALS—ALSFRS-R					
	AUC (95% CI)	Optimal cutoff (95% CI)	Sensitivity (%)	Specificity (%)	*p*
MRR (ms^–1^)	0.533 (0.366 to 0.694)	0.0053 (0.0031 to 0.0073)	30.77	100	0.779
MRPD (cmH_2_O·ms^–1^)	0.735 (0.570 to 0.863)	−0.300 (−0.535.7 to −0.232.1)	76.92	73.98	<0.001
τ (ms)	0.655 (0.486 to 0.800)	89.08 (48.5 to 147.6)	69.23	61.24	0.094
1/2 RT (ms)	0.506 (0.341 to 0.669)	160 (106 to 206)	76.92	42.31	0.853
CT (ms)	0.648 (0.479 to 0.794)	250 (198 to 282)	76.92	65.38	0.118
SNIP (%pred)	0.618 (0.449 to 0.769)	46.33 (17.4 to 67.0)	76.92	53.85	0.194
FVC (%pred)	0.749 (0.584 to 0.873)	41.7 (35.3 to 106.3)	53.85	92.31	0.009

*ALSFRS-R, amyotrophic lateral sclerosis functional rating scale-revised; AUC, area under curve; CI, confidence interval; MRR, maximum relaxation rate, MRPD, maximum rate of pressure development; τ, tau; 1/2RT, half-relaxation time; CT, contraction time; SNIP, sniff nasal inspiratory pressure; FVC, forced vital capacity; ES, effect size; %pred, percentage of predicted; cmH_2_O, centimeter of water; ALS, amyotrophic lateral sclerosis*.

**Table 5:** Receiver operating characteristic analysis between ALS subjects classified according to a decrease in the respiratory subscore (R-subscore) of the ALSFRS-R scale.

ALS—R-subscore					
	AUC (95% CI)	Optimal cutoff (95% CI)	Sensitivity (%)	Specificity (%)	*p*
MRR (ms^–1^)	0.654 (0.485 to 0.799)	0.0065 (0.0053 to 0.0086)	64.29	72	0.130
MRPD (cmH_2_O ·ms^–1^)	0.721 (0.555 to 0.853)	−0.300 (−0.500 to −0.288)	71.43	72	0.01
τ (ms)	0.824 (0.669 to 0.927)	89.1 (70.1 to 168)	85.71	72	<0.0001
1/2 RT (ms)	0.720 (0.553 to 0.852)	160 (158 to 256)	92.86	52	0.01
CT (ms)	0.657 (0.488 to 0.801)	232 (199 to 280)	78.57	64	0.08
SNIP (%pred)	0.614 (0.445 to 0.765)	67 (60 to 67)	100	32	0.216
FVC (%pred)	0.700 (0.532 to 0.836)	67.5 (63.7 to 106)	85.71	52	0.03

*AUC, area under curve; CI, confidence interval; MRR, maximum relaxation rate, MRPD, maximum rate of pressure development; τ, tau; 1/2RT, half-relaxation time; CT, contraction time; SNIP, sniff nasal inspiratory pressure; FVC, forced vital capacity; ES, effect size; %pred, percentage of predicted; cmH_2_O, centimeter of water; ALS, amyotrophic lateral sclerosis*.

**Table 6:** Receiver operating characteristic analysis between ALS subjects classified according to a decrease in FVC.

ALS—FVC_%pred_					
	AUC (95% CI)	Optimal Cutoff (95% CI)	Sensitivity (%)	Specificity (%)	*p*
MRR (ms^–1^)	0.572 (0.485 to 0.799)	0.0086 (0.0053 to 0.0086)	95.83	26.67	0.467
MRPD (cmH_2_O·ms^–1^)	0.781 (0.555 to 0.853)	−0.460 (−0.500 to −0.288)	95.83	53.33	<0.001
τ (ms)	0.794 (0.669 to 0.927)	73.1 (70.1 to 168)	79.17	73.33	0.0001
1/2 RT (ms)	0.632 (0.553 to 0.852)	174 (158 to 256)	62.50	73.33	0.162
CT (ms)	0.536 (0.488 to 0.801)	304 (199 to 280)	100	13.33	0.713
SNIP (%pred)	0.769 (0.445 to 0.765)	46.7 (60 to 67)	79.17	73.33	0.002

*AUC, area under curve; CI, confidence interval; MRR, maximum relaxation rate, MRPD, maximum rate of pressure development; τ, tau; 1/2RT, half-relaxation time; CT, contraction time; SNIP, sniff nasal inspiratory pressure; FVC, forced vital capacity; ES, effect size; %pred, percentage of predicted; cmH_2_O, centimeter of water; ALS, amyotrophic lateral sclerosis*.

## Discussion

The main findings of this study are that (1) the sniff test provides parameters, apart from its peak pressure, able to discriminate between healthy and middle stage ALS subjects, and that (2) some of these parameters, namely τ, MRPD, and 1/2RT, are more sensitive in detecting impaired inspiratory muscle function in ALS than the peak pressure itself.

According to Kyroussis et al. (22), measurements of relaxation rates obtained from nasal sniffs accurately reflects those from esophageal pressure curves and can be used as an index of the onset and recovery of respiratory muscle fatigue. Moreover, measurements of nasal sniffs are simple, tolerated, and minimally invasive and can provide a quantitative response index to fatigue and therapeutic interventions in neuromuscular disease patients (47, 48). In our study, all parameters derived from the SNIP curve were significantly different between middle stage ALS and healthy subjects, being in agreement with two previous studies performed in subjects with neuromuscular disorders (47, 49). Evangelista et al. (49), observed that a reduced MRR was reliable in identifying the delayed relaxation of the respiratory muscles in myotonic dystrophy type 1 patients when compared to healthy controls; while Garcia-Rio et al. (47), despite heterogeneity of the study population, found that the decreased MRR of neuromuscular disease patients was accompanied by the deterioration in the functional reserve of the diaphragm as well as of the inspiratory muscles.

The rationale for measuring relaxation rates from pressure curves is based on the assumption that the decay portion of the curve, when expiration is totally passive, corresponds to the relaxation phase of inspiratory muscle contraction (18). The decrease in MRR and increase in τ are adaptive mechanisms and had been shown to be an early sign of the onset of fatigue (17, 50). The alterations of these parameters occur before the decrease in peak diaphragmatic pressure (17, 31, 47). When respiratory muscles do develop fatigue the peak pressure decreases linearly with the slowing of the MRR and exponentially with the increase of τ due to common or concomitant metabolic changes of the muscle fiber (17, 34, 51). In addition, the loss of muscular force during fatigue makes the muscle contractile speed to decrease resulting in an increase in CT and prolongation of relaxation time as an adaptive mechanism (52, 53) which is also related to intracellular and metabolic factors (i.e., the decline of the calcium uptake from the sarcoplasmic reticulum, depletion of ATP, and intracellular acidosis) (48, 54).

To our knowledge, apart from various studies about the relaxation rate in healthy adults (21, 23, 33) and different diseases [COPD (51), cystic fibrosis (55, 56), and intubated patients weaning from mechanical ventilation (31)], the literature is scarce about the measurements of MRPD, τ, CT, and 1/2RT in neuromuscular disease patients precluding the possibility of comparing our data to data derived from a similar population. Our results showed a decreased MRPD in middle stage ALS when compared to healthy subjects and, as it is derived from the initial incline of the SNIP curve and reflects respiratory muscle function (11) as well as respiratory motor output (19), we believe that this parameter is linked to the decreased capacity of the diaphragm to generate force and expand the lungs (39). Furthermore, as the already weakened respiratory muscles of patients with ALS are easily fatigable (14), the results found about the contractile properties (CT and MRPD) and dynamics of relaxation (MRR, τ, and 1/2RT) of the diaphragm (11, 16) indicates a high respiratory muscle load and reinforces the hypothesis that the middle stage ALS subjects were presumably at risk of developing respiratory muscle fatigue (14, 18, 21, 33, 56).

The ALSFRS-R is a simple and reliable scale that predicts survival and can be used as the only functional outcome measure in early phase trials (40), while its R-subscore was designed to assess indirectly the respiratory function (36) being also sensitive in detecting early respiratory symptoms of ventilatory insufficiency (57–59). Castrillo-Viguera et al. (41) suggested that a percentage change of at least 20–25% in the slope of decline of the ALSFRS-R scale would represent a clinically meaningful effect. Because of this, we chose to subdivide the ALS subjects into those with ALSFRS-R of ≤30 and >30 points (decline of 15 points—37.5%) and with the R-subscore of ≤9 and >9 points (decline of 3 points—25%). Moreover, as changes in FVC_%pred_ over time strongly predicts respiratory muscle weakness, ventilatory failure and death in ALS (2, 38, 39), subjects were also subdivided into ≤70 and >70% FVC_%pred_ subgroups.

The value of the FVC_%pred_ was the only parameter that differed between middle stage ALS subjects of both ALSFRS-R and R-subscore subgroups, possibly because the decrease of this parameter is not related only to respiratory musculature function (36, 60). On the other hand, when subdividing according to the R-subscore, 1/2RT, and τ values were significantly different between middle stage ALS subjects which demonstrate that these diaphragmatic properties (32, 61) are probably related to the respiratory function assessed by this subdomain. Presumably, the most interesting fact is that SNIP_%pred_, a parameter that reflects the diaphragmatic strength and predicts survival in ALS (62), only differ between those middle stage ALS subjects classified according to the decline in FVC_%pred_. Although data were collected in a single point of the disease stage, it is known that the peak pressure of sniff test declines less when compared to the decline in ALSFRS-R (8) leading us to consider that SNIP_%pred_ is not a parameter that is sensitive to small changes in the ALSFRS-R and R-subscore. Regarding MRPD and τ, the results were not surprising since the first is related to respiratory muscle function (11) as well as related to neural adaptations (19, 20, 63) and the second increases well before diaphragmatic pressure is reduced during respiratory muscle weakness or fatigue (17, 34).

The results of the ROC curves show that all parameters extracted from the sniff curve can highly discriminate middle stage ALS from healthy subjects. When taking into account the functional decline of ALS subjects, only MRPD and FVC_%pred_ could predict a fall in 37.5% of the ALSFRS-R score. Among all parameters, τ provides the highest discriminative power in predicting a decline of 25% in the R-subscore. This power was even higher than FVC_%pred_, possibly because the R-subscore is less sensitive in predicting a fall in FVC_%pred_ (57). Moreover, as ALS patients with R-subscore <11 points are considered with relevant symptoms of respiratory distress as well as at risk of respiratory insufficiency (57, 58) and peak pressure of sniff test could not detect a fall in the ALSFRS-R and R-subscore, we believe that the SNIP_%pred_ might not be a parameter as reliable as some parameters extracted from the SNIP curve (i.e., τ, MRPD, and 1/2RT) or FVC_%pred_ in detecting a clinically meaningful decline in functional and respiratory status. The SNIP_%pred_ was reliable in detecting respiratory muscle weakness (39, 60) in our middle stage ALS subjects only when considering the FVC_%pred_ classification; nevertheless, MRPD and τ parameters were still more sensitive than SNIP_%pred_.

It is unlikely that the results found are investigator related since all measurements were performed by the same experienced respiratory physiotherapist. We believe that four are the main limitations of the study. First, even with a calculated statistical power of 1 − β = 0.99, our ALS cohort may be limited in terms of sample size; second, the mean age of ALS included is lower than those of the main epidemiological studies (64, 65); third, we included only ALS patients at middle stage of the disease; and fourth, not all subjects could be paired by the same exact age and BMI. Further studies including patients with other motor neuron disorders are needed. Finally, ongoing longitudinal studies are already investigating these parameters during varying levels of disease progression in order to identify differences between patients with and without the need for non-invasive ventilation as well as the optimal parameter and its cutoff point able to predict an appropriate timing for the initiation of non-invasive ventilation.

In terms of clinical applicability, the calculation of the SNIP curve parameters can be easily performed and give more information about the state of the respiratory muscles, thus possibly allowing an early detection of weakness or fatigue before respiratory failure is reached (35, 53) as well as early implementation of new therapeutic interventions before the beginning of the peak pressure decay of the SNIP curve (17, 31, 51).

## Conclusion

The contractile properties and relaxation rates of the diaphragm are altered in middle stage spinal onset ALS when compared with healthy subjects. When assessed through the nasal inspiratory sniff test, these parameters are able to discriminate between those ALS subjects with early decline in inspiratory muscle function and those who not. In addition, despite the limitations of our cohort and especially the lack of longitudinal data, we suggest that τ, MRPD, and 1/2RT parameters may be able to predict ALS patients at risk of ventilatory failure before the beginning of the fall in peak pressure of sniff test.

## Ethics Statement

All procedures performed in studies involving human participants were in accordance with the recommendations of the National Health Council in Brazil according to the resolution 466/12. The protocol was approved by the Research Ethics Committee of the Hospital Universitário Onofre Lopes under number 1.344.512/2015. All subjects gave written informed consent in accordance with the Declaration of Helsinki.

## Author Contributions

AS, GF, and VR designed the study. GF, VR, and AA supervised the study. AS, LM, and MD-J collected the data and performed clinical assessment. AS, GF, FP, VR, and AA analyzed/interpreted the data. AS wrote the manuscript, and GF, LM, FP, MD-J, VR, and AA revised the manuscript.

## Conflict of Interest Statement

The authors declare that the research was conducted in the absence of any commercial or financial relationships that could be construed as a potential conflict of interest.
