# Supplementary material for: Multiparametric Analysis of Sniff Nasal Inspiratory Pressure Test in Middle Stage Amyotrophic Lateral Sclerosis
Source: Front Neurol. 2018 May 2;9:306. doi: 10.3389/fneur.2018.00306 (PMC5940741; doi:10.3389/fneur.2018.00306)
Supplement: Supplementary file 1 [file Table_1.docx]

**SUPPLEMENTARY MATERIAL IN DEPTH OF STATISTICAL ANALYSIS AND RESULTS**

**STATISTICAL ANALYSIS**

For parametric data, effect sizes were calculated using Cohen’s *d* for intergroup analysis and interpreted as small (<0.50), moderate (between 0.50 and 0.80), and large (>0.80)] and Cohen’s *f* for subgroup analysis as small (<0.25), moderate (between 0.25 and 0.40), and large (>0.40)].([1](#_ENREF_1)) For non-parametric data, Cohen's *d* was calculated for intergroup analysis and interpreted as small (<0.10), moderate (between 0.10 and 0.30), and large (>0.50)([2](#_ENREF_2)) and ɛ^2^ for subgroup analysis as small (<0.06), moderate (between 0.06 and 0.14), and large (>0.14)] ([3](#_ENREF_3), [4](#_ENREF_4)).

**RESULTS**

|  | **Diagnosis** | **Region of onset** | **Distal or proximal** | **Phenotype** | **Familial ALS** | **Cognitive impairment** |
| --- | --- | --- | --- | --- | --- | --- |
| #1 | Definitive | Left upper limb | Distal | Classic | No | No |
| #2 | Probable | Right upper limb | Proximal | Classic | No | No |
| #3 | Definitive | Right lower limb | Proximal | Classic | No | No |
| #4 | Probable | Right upper limb | Distal | Classic | No | No |
| #5 | Probable | Left lower limb | Distal | Primary lateral sclerosis | No | No |
| #6 | Definitive | Left upper limb | Proximal | Classic | No | No |
| #7 | Definitive | Lower limbs | Distal | Flail legs | No | No |
| #8 | Probable | Right upper limb | Distal | Classic | No | No |
| #9 | Definitive | Right lower limb | Proximal | Classic | No | No |
| #10 | Definitive | Left lower limb | Distal | Classic | No | No |
| #11 | Probable | Lower limbs | Proximal | Flail legs | No | No |
| #12 | Definitive | Right upper limb | Distal | Classic | No | No |
| #13 | Probable | Lower limbs | Distal | Flail legs | No | No |
| #14 | Definitive | Right upper limb | Distal | Classic | No | No |
| #15 | Definitive | Right lower limb | Distal | Classic | No | No |
| #16 | Probable | Left lower limb | Proximal | Classic | No | No |
| #17 | Probable | Upper limbs | Distal | Primary lateral sclerosis | No | No |
| #18 | Definiive | Left lower limb | Distal | Classic | No | No |
| #19 | Probable | Right lower limb | Distal | Classic | No | No |
| #20 | Probable | Lower limbs | Distal | Flail legs | No | No |
| #21 | Definitive | Right upper limb | Distal | Classic | No | No |
| #22 | Definitive | Right upper limb | Distal | Classic | No | No |
| #23 | Definitive | Right upper limb | Distal | Progressive muscular atrophy | No | No |
| #24 | Probable | Right lower limb | Distal | Classic | No | No |
| #25 | Definitive | Left upper limb | Distal | Classic | No | No |
| #26 | Probable | Lower limbs | Proximal | Classis | No | No |
| #27 | Probable | Right lower limb | Distal | Polyneuritic pattern | No | No |
| #28 | Probable | Upper limbs | Distal | Classis | No | No |
| #29 | Probable | Right lower limb | Distal | Classic | No | No |
| #30 | Definitive | Right upper limb | Distal | Classic | No | No |
| #31 | Probable | Right lower limb | Distal | Classic | No | No |
| #32 | Probable | Right lower limb | Distal | Classic | No | No |
| #33 | Probable | Left upper limb | Distal | Primary lateral sclerosis | No | No |
| #34 | Probable | Left upper limb | Distal | Classic | No | No |
| #35 | Probable | Lower limbs | Distal | Progressive muscular atrophy | No | No |
| #36 | Probable | Right upper limb | Distal | Primary lateral sclerosis | No | No |
| #37 | Probable | Right upper limb | Distal | Polyneuritic pattern | No | No |
| #38 | Definitive | Right upper limb | Distal | Progressive muscular atrophy | No | No |
| #39 | Probable | Right upper limb | Distal | Progressive muscular atrophy | No | No |

**Supplementary table S1**. Diagnosis criteria, region of onset, local of muscle weakness symptoms onset, clinical phenotype as well as the presence of familial ALS and cognitive impairment of all ALS subjects included in the study. All parameters shown were assessed by a neurologist.

| Subjects | **Clinical Stage** | **Amyotrophic Lateral Sclerosis Functional Rating Scale-revised** | | | | | | | | | | | |
| --- | --- | --- | --- | --- | --- | --- | --- | --- | --- | --- | --- | --- | --- |
|  |  | Speech | Salivation | Swallowing | Handwriting | Cutting food | Dressing and hygiene | Turning in bed and adjusting bed clothes | Walking | Climbing stairs | Dyspnea**^*^** | Orthopnea**^*^** | Respiratory insufficiency**^*^** |
| #1 | 3 | 4 | 4 | 4 | 1 | 1 | 2 | 3 | 3 | 1 | 3 | 4 | 4 |
| #2 | 3 | 4 | 2 | 4 | 3 | 3 | 3 | 3 | 3 | 1 | 3 | 3 | 4 |
| #3 | 3 | 4 | 4 | 4 | 4 | 2 | 2 | 3 | 3 | 3 | 3 | 3 | 4 |
| #4 | 3 | 4 | 4 | 4 | 4 | 1 | 3 | 2 | 2 | 1 | 3 | 3 | 4 |
| #5 | 3 | 3 | 4 | 4 | 3 | 3 | 2 | 2 | 1 | 0 | 4 | 4 | 4 |
| #6 | 3 | 4 | 1 | 2 | 4 | 0 | 1 | 3 | 4 | 4 | 1 | 3 | 4 |
| #7 | 3 | 2 | 3 | 3 | 1 | 2 | 3 | 3 | 3 | 3 | 4 | 4 | 4 |
| #8 | 3 | 2 | 4 | 2 | 0 | 0 | 0 | 0 | 1 | 0 | 2 | 3 | 4 |
| #9 | 3 | 4 | 4 | 4 | 2 | 1 | 1 | 2 | 2 | 1 | 4 | 4 | 4 |
| #10 | 3 | 2 | 4 | 3 | 0 | 0 | 0 | 2 | 1 | 0 | 3 | 4 | 4 |
| #11 | 3 | 1 | 0 | 0 | 3 | 2 | 2 | 1 | 2 | 0 | 2 | 2 | 4 |
| #12 | 3 | 3 | 2 | 2 | 0 | 0 | 0 | 0 | 0 | 0 | 2 | 3 | 4 |
| #13 | 3 | 4 | 4 | 3 | 4 | 4 | 4 | 3 | 3 | 2 | 4 | 4 | 4 |
| #14 | 3 | 4 | 4 | 4 | 4 | 4 | 4 | 4 | 4 | 4 | 3 | 3 | 4 |
| #15 | 3 | 3 | 4 | 3 | 3 | 3 | 3 | 4 | 2 | 1 | 4 | 4 | 4 |
| #16 | 3 | 3 | 3 | 3 | 3 | 1 | 2 | 1 | 1 | 0 | 2 | 2 | 2 |
| #17 | 3 | 2 | 3 | 3 | 2 | 3 | 3 | 3 | 3 | 3 | 3 | 4 | 4 |
| #18 | 3 | 1 | 3 | 2 | 0 | 0 | 0 | 0 | 0 | 0 | 2 | 2 | 4 |
| #19 | 3 | 1 | 3 | 3 | 3 | 3 | 2 | 2 | 1 | 0 | 2 | 3 | 4 |
| #20 | 3 | 4 | 4 | 4 | 2 | 2 | 0 | 0 | 0 | 4 | 4 | 4 | 4 |
| #21 | 3 | 2 | 3 | 3 | 2 | 1 | 3 | 3 | 2 | 1 | 3 | 4 | 4 |
| #22 | 3 | 3 | 3 | 4 | 3 | 3 | 2 | 3 | 4 | 4 | 3 | 2 | 4 |
| #23 | 3 | 3 | 4 | 3 | 3 | 1 | 1 | 1 | 2 | 0 | 1 | 2 | 2 |
| #24 | 3 | 1 | 0 | 0 | 3 | 2 | 2 | 1 | 2 | 0 | 2 | 2 | 4 |
| #25 | 3 | 4 | 4 | 4 | 4 | 4 | 3 | 3 | 1 | 0 | 2 | 3 | 4 |
| #26 | 3 | 3 | 3 | 3 | 4 | 4 | 4 | 4 | 4 | 3 | 3 | 4 | 4 |
| #27 | 3 | 3 | 4 | 3 | 3 | 1 | 2 | 1 | 1 | 0 | 2 | 1 | 2 |
| #28 | 3 | 4 | 4 | 3 | 2 | 0 | 0 | 2 | 3 | 0 | 1 | 2 | 4 |
| #29 | 3 | 2 | 3 | 2 | 3 | 1 | 1 | 1 | 1 | 0 | 4 | 4 | 4 |
| #30 | 3 | 3 | 2 | 2 | 3 | 3 | 3 | 2 | 2 | 1 | 2 | 3 | 4 |
| #31 | 3 | 4 | 4 | 4 | 3 | 1 | 2 | 3 | 2 | 0 | 4 | 4 | 4 |
| #32 | 3 | 4 | 4 | 4 | 4 | 4 | 3 | 4 | 3 | 2 | 4 | 4 | 4 |
| #33 | 3 | 2 | 3 | 2 | 4 | 2 | 3 | 4 | 3 | 3 | 3 | 4 | 4 |
| #34 | 3 | 4 | 4 | 4 | 3 | 3 | 2 | 2 | 0 | 0 | 4 | 4 | 4 |
| #35 | 3 | 4 | 4 | 4 | 4 | 3 | 3 | 3 | 3 | 1 | 2 | 4 | 4 |
| #36 | 3 | 3 | 3 | 3 | 4 | 3 | 4 | 4 | 4 | 3 | 3 | 3 | 4 |
| #37 | 3 | 4 | 4 | 4 | 4 | 3 | 4 | 4 | 4 | 4 | 4 | 4 | 4 |
| #38 | 3 | 3 | 2 | 3 | 3 | 4 | 4 | 3 | 4 | 3 | 4 | 4 | 4 |
| #39 | 3 | 4 | 3 | 4 | 4 | 3 | 4 | 3 | 4 | 3 | 4 | 4 | 4 |

**Supplementary table S2.** Clinical stage and functional capacity of each amyotrophic lateral sclerosis subject of the study according to Roche et al ([5](#_ENREF_5)) and Cerdabaum et al ([6](#_ENREF_6)), respectively. *Respiratory subscore items.

**REFERENCES**

E1. Cohen J. Statistical power analysis for the behavioral sciences. *2ª ed Hillsdale, New Jersey: Lawrence Erbaum* (1988).

E2. Fritz CO, Morris PE, Richler JJ. Effect size estimates: current use, calculations, and interpretation. *Journal of experimental psychology General* (2012) 141(1):2-18. Epub 2011/08/10. doi: 10.1037/a0024338. PubMed PMID: 21823805.

E3. Tomczak M, Tomczak E. The need to report effect size estimates revisited. An overview of some recommended measures of effect size. *Trends in Sport Sciences* (2014) 1(21):19-25.

E4. Olejnik S, Algina J. Measures of Effect Size for Comparative Studies: Applications, Interpretations, and Limitations. *Contemporary educational psychology* (2000) 25(3):241-86. Epub 2000/06/30. doi: 10.1006/ceps.2000.1040. PubMed PMID: 10873373.

E5. Roche JC, Rojas-Garcia R, Scott KM, Scotton W, Ellis CE, Burman R, et al. A proposed staging system for amyotrophic lateral sclerosis. *Brain : a journal of neurology* (2012) 135(Pt 3):847-52. doi: 10.1093/brain/awr351. PubMed PMID: 22271664; PubMed Central PMCID: PMC3286327.

E6. Cedarbaum JM, Stambler N, Malta E, Fuller C, Hilt D, Thurmond B, et al. The ALSFRS-R: a revised ALS functional rating scale that incorporates assessments of respiratory function. *Journal of the neurological sciences* (1999) 169(1):13-21. doi: https://doi.org/10.1016/S0022-510X(99)00210-5.
